# Process integration and future outlook of 2D transistors

**DOI:** 10.1038/s41467-023-41779-5

**Published:** 2023-10-12

**Authors:** Kevin P. O’Brien, Carl H. Naylor, Chelsey Dorow, Kirby Maxey, Ashish Verma Penumatcha, Andrey Vyatskikh, Ting Zhong, Ande Kitamura, Sudarat Lee, Carly Rogan, Wouter Mortelmans, Mahmut Sami Kavrik, Rachel Steinhardt, Pratyush Buragohain, Sourav Dutta, Tristan Tronic, Scott Clendenning, Paul Fischer, Ernisse S. Putna, Marko Radosavljevic, Matt Metz, Uygar Avci

**Affiliations:** Intel Corporation, Components Research, Hillsboro, OR USA

**Keywords:** Electronic devices, Two-dimensional materials

## Abstract

2D semiconductors have been proposed as a potential option to replace or complement silicon electronics at the nanoscale. Here, the authors discuss the recent progress and remaining challenges that need to be addressed by the academic and industrial research communities towards the commercialization of 2D transistors.

## Teaser

The academic and industrial communities have proposed two-dimensional (2D) transition metal dichalcogenide (TMD) semiconductors as a future option to supplant silicon transistors at sub-10nm physical gate lengths. In this Comment, we share the recent progress in the fabrication of complementary metal-oxide-semiconductor (CMOS) devices based on stacked 2D TMD nanoribbons and specifically highlight issues that still need to be resolved by the 2D community in five crucial research areas: contacts, channel growth, gate oxide, variability, and doping. While 2D TMD transistors have great potential, more research is needed to understand the physical interactions of 2D materials at the atomic scale.

**Fig. 1:**
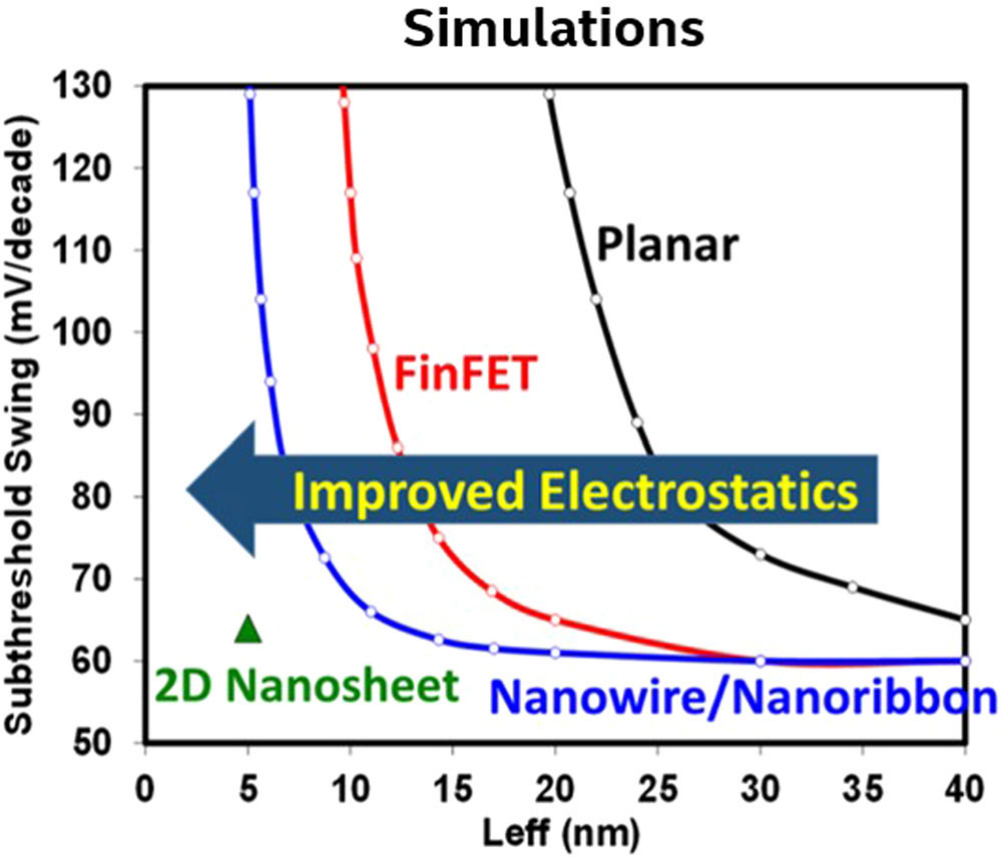
Comparisons of subthreshold swing (SS) versus channel length for various transistor geometries illustrating the advantage of 2D nanosheets. The simulation of SS for semiconductor geometries (Planar, FinFET, nanoribbon, and 2D nanosheet) versus Leff (gate length) shows competitive electrostatics of 2D transition metal dichalcogenides compared to Si at sub-10 nm physical gate lengths. The 2D nanosheet SS is calculated using atomistic density functional theory, whereas the other geometries are from electrostatic solutions.

The academic and industrial communities have proposed 2D TMD semiconductors (i.e., MoS_2_, WSe_2_, etc.) as potential alternatives to silicon (Si) transistors at sub-10 nm physical gate lengths with Gate All Around (GAA) stacked nanoribbons (NRs)^1–4^. Electrostatics of GAA NRs dictate that, as physical gate-lengths shrink below 10 nm, the thickness of the semiconductor channel must decrease well below 5 nm to maintain a sub 70 mV/dec subthreshold swing (SS) and thus turn off the transistor effectively. Without the low SS, the transistor would either leak too much current in the off state or require higher voltages to operate. We illustrate this point in Fig. 1 by comparing the calculated SS, using density functional theory (DFT) atomistic simulations for the 2D nanosheet, for various device geometries. At sub-10 nm gate lengths, direct tunneling between source and drain is expected to result in a high off-state leakage for Si and other traditional bulk transistors. The large band gap of TMDs combined with their high effective mass suppresses direct source-to-drain tunneling and keeps the off-state current low. Furthermore, Si mobility decreases with smaller channel thickness, whereas mobilities of 2D TMDs are maintained^5^. This presents an opportunity for 2D TMD transistors to potentially replace Si at sub-10 nm physical gate lengths or rather in sub-1 nm nodes. However, the practical feasibility and timing of this potential transition to new semiconductor materials remain open questions. Many challenges need to be resolved by the 2D community before Si can be replaced. In this Comment, we lay out some of the promising properties of 2D TMDs and highlight the current issues preventing their adoption by the semiconductor industry.

**Fig. 2:**
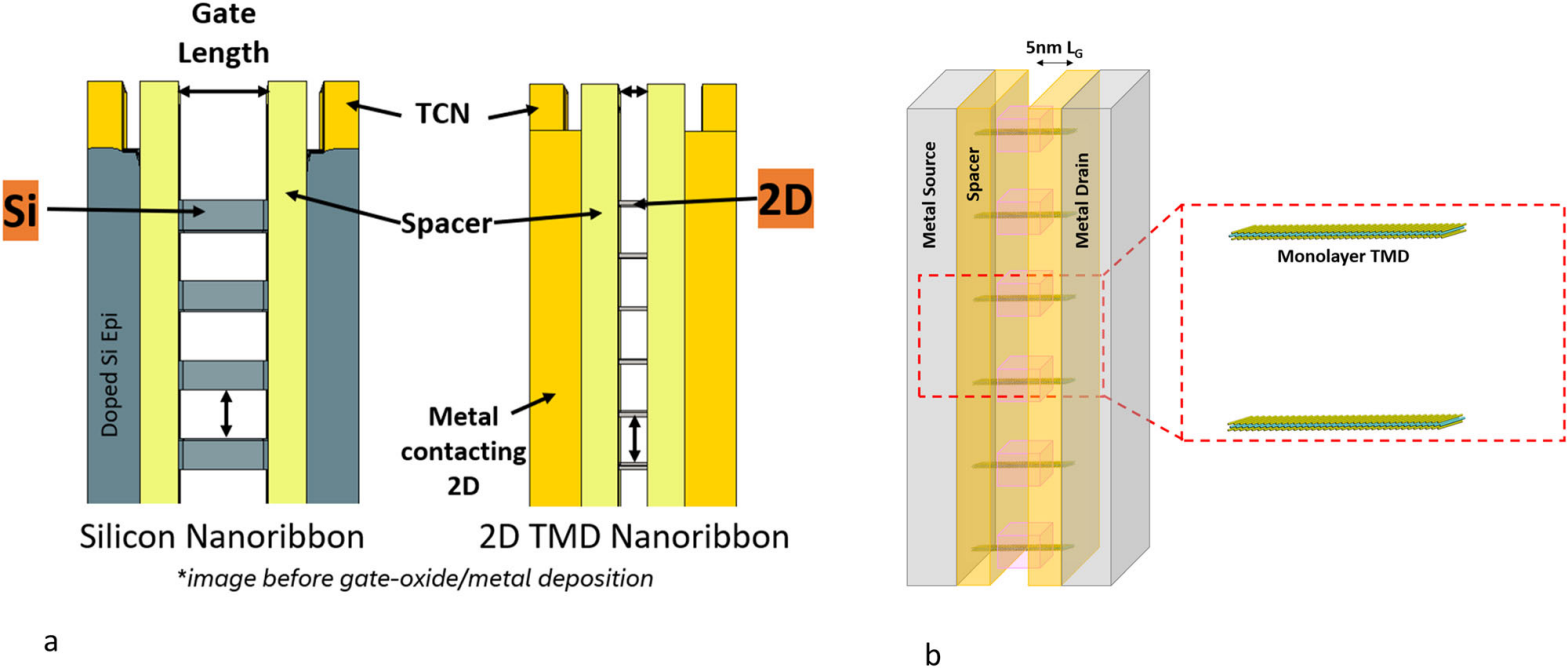
Illustrations of stacked nanoribbon transistors with the same physical height. **a** Cross-section image of a stacked gate-all-around (GAA) Si compared to stacked GAA 2D TMD nanoribbon (NR) transistors with the same physical height. Assuming the same volume for gate oxide and gate metal deposition, four stacked Si NR would have the same height as six stacked 2D TMD NR. Therefore, 2D TMD drive currents need only be 2/3 of Si drive currents to be competitive. Panel **b** represents the same drawing but at the scale of individual atoms for the 2D TMD films. Metal contacts are represented in grey, the spacer dielectric is represented in yellow and the gate oxide is represented in pink. The gate metal is not represented. © IEEE (2023). Figure 2a reprinted with minor modifications, with permission, from K. P. O’Brien et al., “Advancing 2D monolayer CMOS through contact, channel and interface engineering,” 2021 IEEE International Electron Devices Meeting (IEDM), San Francisco, CA, USA, 2021, pp. 7.1.1–7.1.4, 10.1109/IEDM19574.2021.9720651.

The future of semiconductor devices centers around stacked GAA NR structures, and 2D TMDs provide a unique scaling advantage over Si GAA NRs. Figure 2 shows the cross-section of a stacked Si GAA NR and a stacked 2D TMD GAA NR transistor at equivalent heights to account for common process integration considerations, including challenges in etching the transistor stack. There is a clear benefit to the 2D TMD GAA NR structure since more 2D TMD NRs can be placed in the same volume. Specifically, the same gate height can accommodate six 2D TMD NRs compared to only four Si NRs. This enables a 2D TMD GAA NR transistor of the same dimensions to achieve higher performance than Si even if a single TMD nanosheet has lower performance than an individual Si nanosheet.

## Fabrication challenges for 2D TMD transistors

**Fig. 3:**
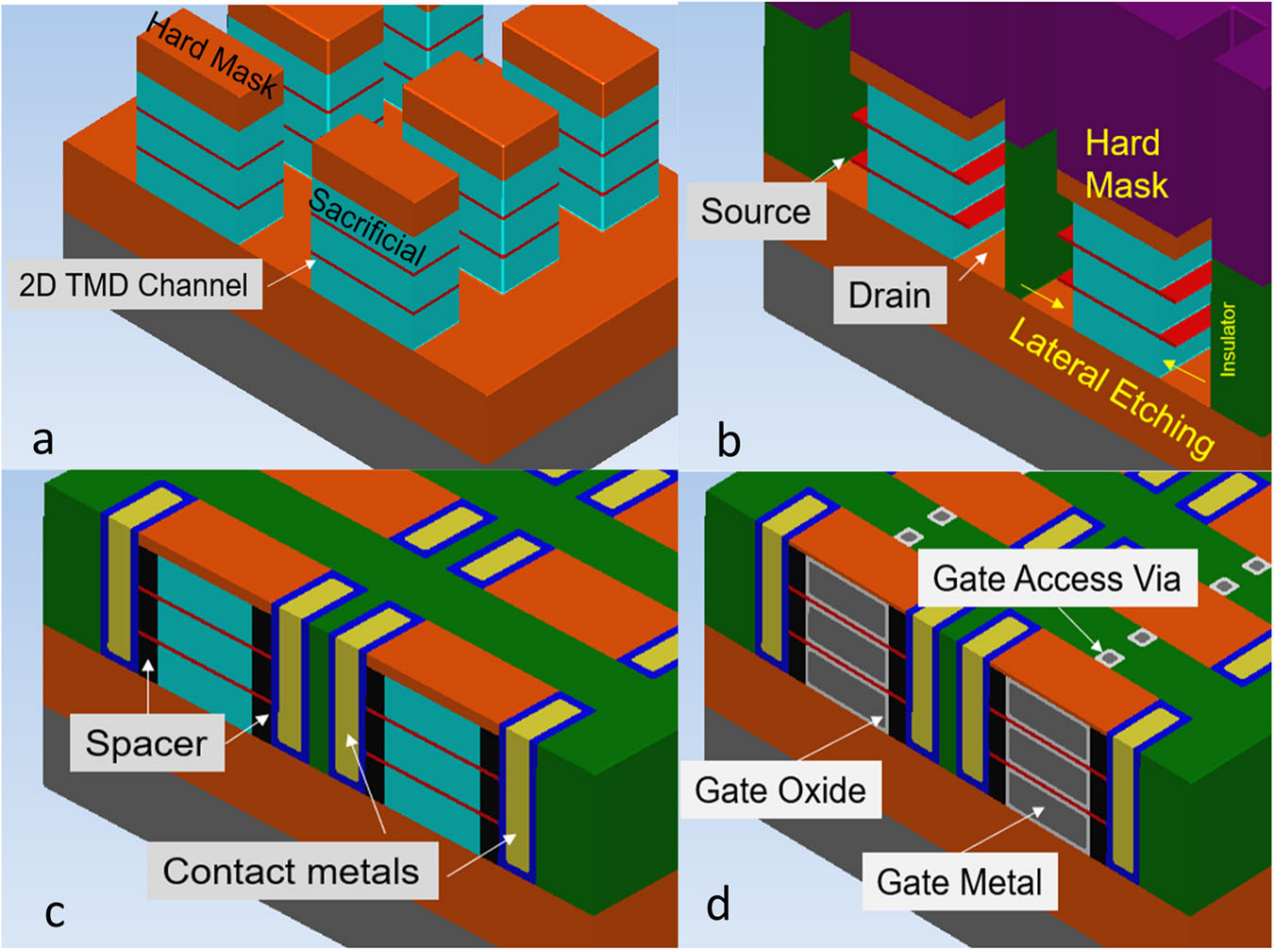
Simplified 2D TMD stacked NR integration scheme with the 2D TMD deposited or transferred onto a sacrificial oxide. We restrict the image to only include two stacked NRs for illustrative purposes. The red colour represents the TMD, whereas the light blue colour represents a sacrificial film (which can be an oxide or another material). **a** Etching of the stacked 2D TMD NRs. **b** Lateral etching opening up the source and drain contact regions to form the inner spacer. 2D TMDs under the spacer must be doped for the transistor to operate properly. **c** Formation of metal contacts (blue and yellow layers) by a damascene process. Further, it is possible to recess the spacer layer (black) to expose a wider region of the TMD film and enable non-edge contacts. **d** Gate formation by etching access via to fill the gate oxide (light grey) and metal (dark grey) with an atomic layer deposition (ALD) process. The reader is cautioned that gate oxide depositions present unique challenges due to the nature of 2D TMD van der Waals materials. Typical ALD processes depend on dangling bonds for material nucleation but defect-free 2D TMDs do not have dangling bonds.

Fabrication of 2D TMD NR transistors must include the following key process steps: (1) deposition of the 2D TMDs, (2) formation of the stacked NR channels, (3) doping in the spacer region, and (4) deposition of the source/drain contact regions and the gate oxide/ metal regions. In Fig. 3 we share a simplified 2D TMD stacked NR integration scheme to draw the reader’s attention to the challenges underreported in current literature. In Fig. 3a we show an image of a cut 2D stacked NR structure with a sacrificial oxide layer separating the 2D sheets, revealing the first of many challenges during the fabrication process, i.e., the impact of etching on the 2D channel performance. In the final product, since the width of the NRs could be as small as 5–10 nm, the defects at the edge of the NRs and the edge termination may impact the device’s performance. In Fig. 3b, we illustrate the formation of the spacer region using a directional and isotropic etch. Many of the etching solutions used in modern Si technologies do not translate to 2D TMD stacked NR structures, and the interaction of dry, wet and vapor etch chemistries with 2D TMDs is still poorly understood. Therefore, selective chemistries that remove surrounding materials without harming TMD monolayers are needed.

In Fig. 3c and d, we show the formation of the source/drain contacts and the gate oxide/metal depositions. To form the source/drain contact regions, two crucial factors must be considered: the area of the exposed 2D film and the quality of the exposed material, which may be altered by the etching process. While specific fabrication schemes can expose larger areas of the 2D film in the contact region, they may also degrade the 2D TMD surface, thus increasing the contact resistance or causing mechanical instabilities. Other challenges without clear solutions are how to dope the 2D film under the spacer and whether the best option will rely on substitutional doping or remote charge doping.

There is a serious concern regarding the mechanical robustness of the monolayer films because the TMDs are supported only on 2 or 3 sides at multiple stages of the fabrication process. For example, in Fig. 3b the TMD layers are only supported by the green insulating layer on two sides and by the blue sacrificial oxide layer on 1 side, leaving one side open to air prior to the inner spacer deposition. Fortunately, TMDs are characterized by a robust Young’s modulus of ~270 MPa in monolayer MoS_2_^6^, compared to 130–188 MPa of Si^7^. During the fabrication process, the impact of internal stresses and thermal expansion of the surrounding films can be an issue, along with capillary collapse if wet etches are used in the process. These considerations highlight the need for more non-invasive solutions to etch films surrounding TMDs without damaging their properties. A mitigation strategy would be to fabricate the TMD nanoribbons with a sacrificial backing layer to provide mechanical support.

Doping the contact region and under the spacer layer will also be a challenge in future TMD devices. There are currently two generic methods to dope TMD materials, either by substitutional doping or remote charge doping. In the past, substitutional doping in Si was implemented by high-energy implantation of dopant atoms into the Si lattice. This approach, however, cannot be applied to a stacked TMD nanoribbon because of the monolayer nature of TMDs. Substitutional doping requires either regrowth of the TMD films or extremely complicated fabrication techniques. Another unique possibility for TMDs is to place dipole charges near the channel to effectively dope the areas of interest. Yet, this strategy may introduce potential coulomb scattering from the very same dipoles, requiring further adjustments.

As with Si, the choice of the nanoribbon stack architecture has a profound impact on multiple areas and design decisions^8^. In Fig. 3 we shared a method to fabricate a stacked nanoribbon structure based on a single type of TMD acting as semiconducting channels for both NMOS and PMOS devices. For the NMOS stacked on the PMOS route, it may be possible to use separate materials for NMOS and PMOS transistors. The optimal design of these structures remains an open question because of trade-offs between drive current and cell capacitance.

## Deposition of a gate oxide on a 2D TMD

**Fig. 4:**
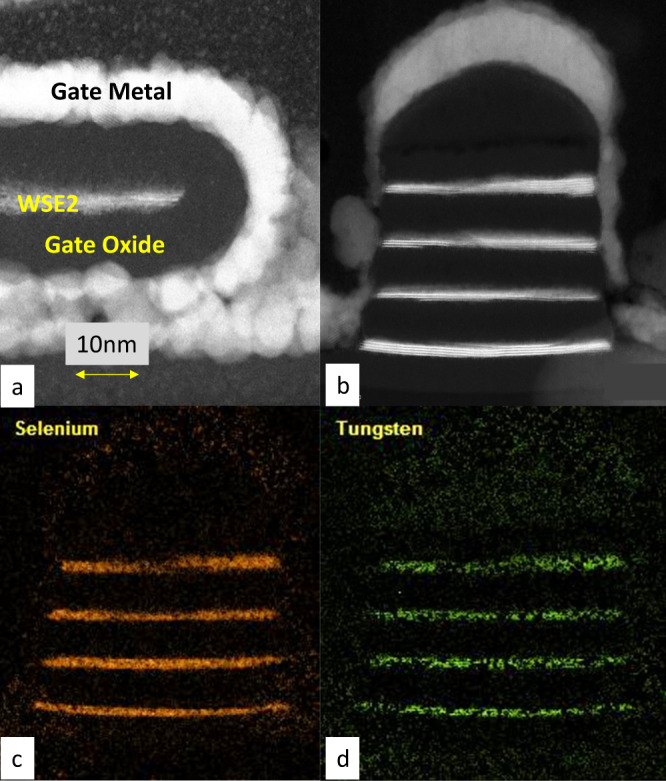
Transmission electron microscopy (TEM) cross-section images of 2D TMD nanoribbons. **a** 2D TMD NR with GAA oxide and metal, with 10nm size bar. **b**–**d** Four stacked 2D WSe2 NRs with Se and W TEM HAADF signals.

Depositing a gate oxide that meets stringent reliability and equivalent oxide thickness (EOT) requirements remains a continued research subject^9,10^. For example, the gate oxide thickness needs to scale to an electrical EOT of ~1 nm while surviving oxide breakdown requirements, and a physical thickness of <3 nm based on volume constraints. Figure 3d shows an example of gate oxide/metal deposition. 2D TMDs are van der Waals materials with no dangling bonds. As atomic layer deposition (ALD) relies on dangling bonds to initiate the growth and controllably synthesize a finite dielectric thickness, the ALD growth of gate oxides on 2D TMD surfaces with minimal defects represents a significant challenge. We have previously reported that physisorption can enhance gate deposition^10^, whereas a strategy of damaging a thicker TMD as a replacement gate may generate unwanted hysteresis and not reach low SS at sub-10 nm gate lengths. In Fig. 4a we show a demonstration of GAA deposition, but the challenges of device integration to obtain the desired final product still need to be addressed. Moreover, the devices may not pass reliability testing. Another aspect of the gate stack technology that remains relatively unexplored is the use of different work-function metals or dipole layers to set multiple NMOS and PMOS threshold voltages. This is a critical requirement if 2D materials are to be used in front-end-of-the-line transistor technology.

## Realistic 2D TMD growth requirements

Although significant progress has been made in the last 10 years on 2D TMD growth quality, research is still needed in this area. One reason Si has survived as the transistor material of choice for over 50 years is the existence of crystalline epitaxial growth processes with extremely low defect densities. 2D TMD growth is currently not at the single crystal level: grain dimensions larger than 10 microns have been demonstrated, but wafer-scale defect-free single-crystalline films are rarely obtained^11^. 2D TMD deposition is usually a stochastic process where the growth initiates at random sites on the wafer, the crystals grow laterally and eventually merge into a polycrystalline material. Due to the stochastic nature of most growth techniques, the random distribution of grain boundaries can cause channel quality variability across different devices. If there are significant differences in the transport properties, the variability in device performance could be too large to meet modern circuit design requirements^2^. In order to circumvent this issue, we have previously introduced methods to selectively grow TMD crystals at deterministic positions, but the approach comes with its own integration challenges^12^. Further developments in 2D TMD deposition techniques to yield coalesced monolayer films with large grain sizes and low defect densities would help to reduce the gap between high-quality Si epitaxy and 2D TMD growth. It is also worth noting that, alternatively, this issue could be a distraction, since the transistor drive current is normally dominated by the contact resistance at sub-10 nm gate lengths. Therefore, the channel mobility variation may have little correlation to the drive current variation.

## Contact resistance

Contact resistance to TMDs is arguably the most crucial problem to solve and the communities have made significant progress over the last few years. That said, NMOS and PMOS contacts to 2D TMDs require continued research if TMDs are to reach a commercial semiconductor product. NMOS contact resistance has made swift progress in the last years with the introduction of semi-metals such as Sb and Bi. While Bi has a low melting point of 271 °C, Sb is preferred due to the ability to survive processing at BEOL temperatures^1,13^. However, it is still unknown whether these semi-metals can meet the rigorous contact resistivity targets at the required physical contact dimensions of 10–20 nm. PMOS contact resistance continues to be an elusive issue. We have found metals such as Ru are good candidates for PMOS WSe_2_ systems if the WSe_2_ is of sufficient quality^1^. 2D material contact resistance remains the Achilles’ heel of 2D TMD performance and we strongly encourage more detailed research in this field.

Since front-end Si transistors are moving to a GAA NR architecture, 2D TMDs will adopt a similar geometry to be competitive. This presents a potential issue because most of the published works trying to address the contact resistance issue are based on evaporated top contacts, whereas the simplest fabrication of an NR transistor tacitly implies the use of an edge contact, as shown in Fig. 3c. It is possible to fabricate a partial wrap-around contact with an ALD metal by changing the etch sequence in Fig. 3c, such that the inner spacer is etched towards the gate more than what is shown in the image. However, this could impact the transistor density. An alternative would be to regrow the TMDs in the contact regions. To this extent, we encourage the scientific community to move towards a GAA architecture, as the future device learnings will provide valuable feedback and enable an acceleration towards a potential integration.

## Future directions

To summarize, we highlighted the reasons why 2D TMD transistors have a chance to replace Si, specifically at sub-10 nm gate lengths. Furthermore, we highlighted areas that need additional academic and industrial research, as well as fundamental understanding, to make this transition a reality. Each of these essential areas, namely (1) the deposition of the 2D TMD, (2) the formation of the stacked NR channels, (3) the doping in the spacer region, and (4) the deposition of both the source/drain region and the gate oxide/metal regions, will need viable manufacturable solutions. In addition, we note that, while the future Si device architecture is based on NRs, today’s body of TMD literature focuses predominantly on back-gated devices. More studies are desperately needed to understand the impact of the NR geometry on the performance of 2D TMD transistors. We must recognize the possibility of this being a strategic inflection point similar to the revolution of Si microelectronics. It is an exciting time to be in the field and we encourage more research to understand the physics and engineering of 2D TMD NR architectures at the atomic scale. Both Academia and Industry need to accelerate the research and learning on these structures, as we are leaving the world of nanotechnology and entering the Angstrom Era. We need to focus on fabricating, controlling, and understanding extremely complex systems where each atom counts.
